# Supplementary material for: Performance of a fully automated plasma tau phosphorylated at threonine 217 immunoassay to reflect amyloid-beta burden in an unselected cohort representative of clinical practice
Source: J Prev Alzheimers Dis. 2026 Mar 25;13(5):100534. doi: 10.1016/j.tjpad.2026.100534 (PMC13053736; doi:10.1016/j.tjpad.2026.100534)
Supplement: Supplementary file 1 [file mmc1.docx]

**Table S1.** Clinical study sites and number of samples (*N* = 588) taken from each site.

| **Clinical site** | **Number of samples** |
| --- | --- |
| Ace Alzheimer Center Barcelona, Spain | 83 |
| Adams Clinical, Watertown, MA, USA | 74 |
| Alzheimer's Research and Treatment Center, Wellington, FL, USA | 92 |
| Barcelonaβeta Brain Research Center, Barcelona, Spain | 18 |
| Charter Research Lady Lake, Lady Lake, FL, USA | 36 |
| Columbus Memory Center, Columbus, GA, USA | 75 |
| Danish Dementia Research Centre, Copenhagen, Denmark | 10 |
| Eastside Research Associates, Redmond, WA, USA | 34 |
| Genesis Neuroscience Clinic, Knoxville, TN, USA | 20 |
| K2 Medical Research Maitland, Maitland, FL, USA | 78 |
| K2 Medical Research, LLC Tampa, Tampa, FL, USA | 55 |
| Scottish Brain Sciences, Edinburgh, UK | 13 |

**Table S2.** ROC-AUC analyses for plasma pTau217 with respect to varying centiloid-based classification cutoffs.

| **Centiloid classification** | **AUC overall  (95 % CI)** | **AUC in SCD  (95 % CI)** | **AUC in MCI and MD  (95 % Cl)** |
| --- | --- | --- | --- |
| 15 | 0.85 (0.814 to 0.887) | 0.791 (0.709 to 0.874) | 0.865 (0.824 to 0.906) |
| 16 | 0.866 (0.832 to 0.9) | 0.81 (0.731 to 0.889) | 0.878 (0.839 to 0.917) |
| 17 | 0.873 (0.839 to 0.907) | 0.817 (0.735 to 0.9) | 0.884 (0.846 to 0.922) |
| 18 | 0.873 (0.839 to 0.907) | 0.816 (0.732 to 0.901) | 0.884 (0.846 to 0.922) |
| 19 | 0.883 (0.851 to 0.916) | 0.847 (0.769 to 0.924) | 0.889 (0.851 to 0.926) |
| 20 | 0.886 (0.853 to 0.919) | 0.847 (0.768 to 0.926) | 0.892 (0.855 to 0.93) |
| 21 | 0.891 (0.859 to 0.923) | 0.854 (0.775 to 0.933) | 0.896 (0.859 to 0.932) |
| 22 | 0.888 (0.856 to 0.921) | 0.845 (0.764 to 0.926) | 0.895 (0.858 to 0.932) |
| 23 | 0.891 (0.859 to 0.924) | 0.862 (0.779 to 0.945) | 0.894 (0.857 to 0.931) |
| 24 | 0.896 (0.864 to 0.928) | 0.866 (0.782 to 0.949) | 0.898 (0.862 to 0.934) |
| 25 | 0.896 (0.864 to 0.928) | 0.866 (0.782 to 0.949) | 0.899 (0.863 to 0.935) |
| 26 | 0.898 (0.866 to 0.93) | 0.866 (0.782 to 0.949) | 0.901 (0.866 to 0.937) |
| 27 | 0.907 (0.877 to 0.938) | 0.866 (0.782 to 0.949) | 0.913 (0.88 to 0.947) |
| 28 | 0.913 (0.883–0.943) | 0.878 (0.796 to 0.961) | 0.918 (0.884 to 0.951) |
| 29 | 0.917 (0.887 to 0.947) | 0.886 (0.801 to 0.971) | 0.92 (0.887 to 0.953) |
| 30 | 0.925 (0.896 to 0.953) | 0.914 (0.847 to 0.982) | 0.924 (0.891 to 0.957) |
| 31 | 0.929 (0.902 to 0.956) | 0.914 (0.847 to 0.982) | 0.929 (0.898 to 0.96) |
| 32 | 0.933 (0.906 to 0.96) | 0.932 (0.872 to 0.993) | 0.931 (0.9 to 0.962) |
| 33 | 0.931 (0.903 to 0.958) | 0.93 (0.863 to 0.997) | 0.927 (0.895 to 0.959) |
| 34 | 0.93 (0.903 to 0.958) | 0.93 (0.863 to 0.997) | 0.927 (0.895 to 0.959) |
| 35 | 0.927 (0.899 to 0.955) | 0.93 (0.863 to 0.997) | 0.922 (0.889 to 0.955) |
| 36 | 0.924 (0.896 to 0.953) | 0.93 (0.863 to 0.997) | 0.918 (0.884 to 0.951) |
| 37 | 0.924 (0.896 to 0.953) | 0.93 (0.863 to 0.997) | 0.918 (0.884 to 0.951) |
| 38 | 0.921 (0.892 to 0.95) | 0.924 (0.854 to 0.994) | 0.915 (0.881 to 0.949) |
| 39 | 0.919 (0.89 to 0.949) | 0.921 (0.849 to 0.994) | 0.913 (0.878 to 0.947) |
| 40 | 0.916 (0.886 to 0.946) | 0.921 (0.849 to 0.994) | 0.907 (0.872 to 0.942) |
| 41 | 0.916 (0.886 to 0.946) | 0.921 (0.849 to 0.994) | 0.907 (0.872 to 0.942) |
| 42 | 0.915 (0.885 to 0.945) | 0.918 (0.842 to 0.994) | 0.907 (0.872 to 0.942) |
| 43 | 0.917 (0.887 to 0.947) | 0.918 (0.842 to 0.994) | 0.909 (0.874 to 0.944) |
| 44 | 0.918 (0.888 to 0.948) | 0.918 (0.842 to 0.994) | 0.911 (0.876 to 0.946) |
| 45 | 0.916 (0.886 to 0.946) | 0.913 (0.834 to 0.992) | 0.909 (0.874 to 0.944) |
| 46 | 0.918 (0.888 to 0.948) | 0.913 (0.834 to 0.992) | 0.912 (0.877 to 0.947) |
| 47 | 0.918 (0.888 to 0.948) | 0.913 (0.834 to 0.992) | 0.912 (0.877 to 0.947) |
| 48 | 0.919 (0.889 to 0.949) | 0.913 (0.834 to 0.992) | 0.913 (0.878 to 0.948) |
| 49 | 0.917 (0.886 to 0.947) | 0.913 (0.834 to 0.992) | 0.91 (0.874 to 0.945) |
| 50 | 0.917 (0.886 to 0.947) | 0.913 (0.834 to 0.992) | 0.91 (0.874 to 0.945) |

The discriminative ability of pTau217 was reported in terms of AUC (CI) for each prespecified centiloid cutoff.
AUC, area under the curve; CI, confidence interval; MCI, mild cognitive impairment;
MD, mild dementia; pTau217, tau phosphorylated at threonine 217; ROC, receiver operating characteristic; SCD, subjective cognitive decline.

**Table S3.** Performance overview at various plasma pTau217 cutoff values with respect to centiloid 24.1.

| **Cutoff, pg/mL** | **PPA (overall), % (95 % CI)** | **NPA (overall), % (95 % CI)** | **PPA (SCD), % (95 % CI)** | **NPA (SCD), %  (95 % CI)** | **PPA (MCI+MD), % (95 % CI)** | **NPA (MCI+MD), % (95 % CI)** |
| --- | --- | --- | --- | --- | --- | --- |
| 0.25 | 89.7 (84.7 to 93.3) | 77.1  (72.7 to 81.0) | 85.3 (69.9 to 93.6) | 81.9 (75.1 to 87.2) | 90.6 (85.1 to 94.2) | 73.7 (67.7 to 79.0) |
| 0.275 | 86.7  (81.2 to 90.7) | 81.2  (77.0 to 84.7) | 82.4 (66.5 to 91.7) | 84.5 (78.0 to 89.4) | 87.5 (81.5 to 91.8) | 78.9 (73.2 to 83.6) |
| 0.3 | 84.6 (78.9 to 89.0) | 85.8 (81.9 to 88.9) | 82.4 (66.5 to 91.7) | 88.4 (82.4 to 92.5) | 85.0 (78.7 to 89.7) | 84.1 (78.8 to 88.2) |
| **0.3165** | **83.1 (77.2 to 87.7)** | **89.8 (86.4 to 92.4)** | **79.4 (63.2 to 89.7)** | **92.3 (87.0 to 95.5)** | **83.8 (77.3 to 88.7)** | **88.4 (83.6 to 91.9)** |
| 0.325 | 80.0 (73.8 to 85.0) | 90.3 (87.0 to 92.9) | 70.6 (53.8 to 83.2) | 92.9 (87.7 to 96.0) | 81.9 (75.2 to 87.1) | 88.8 (84.1 to 92.2) |
| 0.35 | 78.5 (72.2 to 83.7) | 91.6 (88.4 to 94.0) | 64.7 (47.9 to 78.5) | 93.5 (88.5 to 96.5) | 81.3 (74.5 to 86.5) | 90.5 (86.1 to 93.7) |
| 0.375 | 75.9 (69.4 to 81.4) | 93.6 (90.8 to 95.7) | 55.9 (39.5 to 71.1) | 96.1 (91.8 to 98.2) | 80.0 (73.1 to 85.5) | 92.2 (88.1 to 95.0) |
| 0.4 | 72.8 (66.2 to 78.6) | 94.9 (92.3 to 96.7) | 50.0 (34.1 to 65.9) | 96.8 (92.7 to 98.6) | 77.5 (70.4 to 83.3) | 94.0 (90.1 to 96.4) |
| 0.425 | 70.8 (64.0 to 76.7) | 95.4 (92.9 to 97.1) | 50.0 (34.1 to 65.9) | 96.8 (92.7 to 98.6) | 75.0 (67.8 to 81.1) | 94.8 (91.2 to 97.0) |
| 0.45 | 67.2 (60.3 to 73.4) | 95.7 (93.2 to 97.3) | 47.1 (31.5 to 63.3) | 96.8 (92.7 to 98.6) | 71.9 (64.5 to 78.3) | 95.3 (91.7 to 97.3) |
| 0.475 | 64.6 (57.7 to 71.0) | 95.9 (93.5 to 97.5) | 41.2 (26.4 to 57.8) | 96.8 (92.7 to 98.6) | 70.0 (62.5 to 76.6) | 95.7 (92.2 to 97.6) |
| 0.5 | 63.1 (56.1 to 69.5) | 95.9 (93.5 to 97.5) | 41.2 (26.4 to 57.8) | 96.8 (92.7 to 98.6) | 68.1 (60.6 to 74.8) | 95.7 (92.2 to 97.6) |
| 0.525 | 61.0 (54.0 to 67.6) | 95.9 (93.5 to 97.5) | 41.2 (26.4 to 57.8) | 96.8 (92.7 to 98.6) | 65.6 (58.0 to 72.5) | 95.7 (92.2 to 97.6) |
| 0.55 | 57.9 (50.9 to 64.7) | 96.4 (94.1 to 97.9) | 35.3 (21.5 to 52.1) | 97.4 (93.6 to 99.0) | 63.1 (55.4 to 70.2) | 96.1 (92.8 to 97.9) |
| 0.575 | 54.9 (47.9 to 61.7) | 96.4 (94.1 to 97.9) | 32.4 (19.1 to 49.2) | 97.4 (93.6 to 99.0) | 60.0 (52.3 to 67.3) | 96.1 (92.8 to 97.9) |
| 0.6 | 52.3 (45.3 to 59.2) | 96.9 (94.7 to 98.2) | 32.4 (19.1 to 49.2) | 97.4 (93.6 to 99.0) | 56.9 (49.1 to 64.3) | 96.6 (93.3 to 98.2) |
| 0.625 | 46.2 (39.3 to 53.2) | 97.2 (95.1 to 98.4) | 20.6 (10.3 to 36.8) | 97.4 (93.6 to 99.0) | 51.9 (44.2 to 59.5) | 97.0 (93.9 to 98.5) |
| 0.65 | 43.1 (36.3 to 50.1) | 97.5 (95.4 to 98.6) | 20.6 (10.3 to 36.8) | 97.4 (93.6 to 99.0) | 48.1 (40.5 to 55.8) | 97.4 (94.5 to 98.8) |
| 0.675 | 40.0 (33.4 to 47.0) | 98.0 (96.0 to 99.0) | 14.7 (6.45 to 30.1) | 98.1 (94.5 to 99.3) | 45.6 (38.1 to 53.4) | 97.8 (95.1 to 99.1) |
| 0.7 | 35.9 (29.5 to 42.8) | 98.0 (96.0 to 99.0) | 14.7 (6.45 to 30.1) | 98.1 (94.5 to 99.3) | 40.6 (33.3 to 48.4) | 97.8 (95.1 to 99.1) |
| 0.725 | 32.3 (26.1 to 39.2) | 98.0 (96.0 to 99.0) | 14.7 (6.45 to 30.1) | 98.1 (94.5 to 99.3) | 36.3 (29.2 to 43.9) | 97.8 (95.1 to 99.1) |
| 0.75 | 30.3 (24.2 to 37.0) | 98.0 (96.0 to 99.0) | 11.8 (4.67 to 26.6) | 98.1 (94.5 to 99.3) | 34.4 (27.5 to 42.0) | 97.8 (95.1 to 99.1) |
| 0.775 | 27.7 (21.9 to 34.4) | 98.0 (96.0 to 99.0) | 8.82 (3.05 to 23.0) | 98.1 (94.5 to 99.3) | 31.9 (25.2 to 39.4) | 97.8 (95.1 to 99.1) |
| 0.8 | 27.2 (21.4 to 33.8) | 98.7 (97.1 to 99.5) | 8.82 (3.05 to 23.0) | 100.0 (97.6 to 100.0) | 31.3 (24.6 to 38.8) | 97.8 (95.1 to 99.1) |

Youden’s index and associated performance are shown in boldface.
CI, confidence interval; MCI, mild cognitive impairment; MD, mild dementia; NPA, negative percent agreement; PPA, positive percent agreement;
pTau217, tau phosphorylated at threonine 217; SCD, subjective cognitive decline.

**Table S4.** Performance overview at various plasma pTau217 cutoff values with respect to centiloid 30.

| **Cutoff, pg/mL** | **PPA (overall), % (95 % CI)** | **NPA (overall), % (95 % CI)** | **PPA (SCD), % (95 % CI)** | **NPA (SCD), %  (95 % CI)** | **PPA (MCI+MD), % (95 % CI)** | **NPA (MCI+MD), % (95 % CI)** |
| --- | --- | --- | --- | --- | --- | --- |
| 0.25 | 94.4  (90.0 to 96.9) | 76.5 (72.2 to 80.4) | 93.3 (78.7 to 98.2) | 81.8  (75.0 to 87.0) | 94.6 (89.7 to 97.2) | 73.0 (67.1 to 78.1) |
| 0.275 | 92.2  (87.3 to 95.3) | 80.9 (76.8 to 84.4) | 90.0 (74.4 to 96.5) | 84.3  (77.8 to 89.1) | 92.6 (87.2 to 95.8) | 78.7 (73.1 to 83.4) |
| 0.3 | 89.9  (84.7 to 93.5) | 85.3 (81.6 to 88.4) | 90.0 (74.4 to 96.5) | 88.1  (82.1 to 92.2) | 89.9 (84.0 to 93.8) | 83.6 (78.4 to 87.7) |
| **0.319** | **87.7  (82.1 to 91.7)** | **90.2 (87.0 to 92.7)** | **83.3 (66.4 to 92.7)** | **93.1 (88.0 to 96.1)** | **88.5 (82.4 to 92.7)** | **88.5 (83.9 to 91.9)** |
| 0.325 | 86.6  (80.8 to 90.8) | 90.5 (87.2 to 92.9) | 80.0 (62.7 to 90.5) | 93.1 (88.0 to 96.1) | 87.8 (81.6 to 92.2) | 88.9 (84.4 to 92.3) |
| 0.35 | 84.9  (78.9 to 89.4) | 91.7 (88.6 to 94.0) | 73.3 (55.6 to 85.8) | 93.7  (88.8 to 96.5) | 87.2 (80.8 to 91.6) | 90.6 (86.3 to 93.6) |
| 0.375 | 82.1 (75.9 to 87.0) | 93.6  (90.8 to 95.6) | 63.3 (45.5 to 78.1) | 96.2 (92.0 to 98.3) | 85.8 (79.3 to 90.5) | 92.2 (88.2 to 95.0) |
| 0.4 | 78.8 (72.2 to 84.1) | 94.9 (92.3 to 96.6) | 56.7 (39.2 to 72.6) | 96.9 (92.9 to 98.6) | 83.1 (76.3 to 88.3) | 93.9 (90.1 to 96.2) |
| 0.425 | 76.5 (69.8 to 82.1) | 95.4 (92.9 to 97.0) | 56.7 (39.2 to 72.6) | 96.9 (92.9 to 98.6) | 80.4 (73.3 to 86.0) | 94.7 (91.1 to 96.9) |
| 0.45 | 72.6 (65.7 to 78.6) | 95.6 (93.2 to 97.2) | 53.3 (36.1 to 69.8) | 96.9 (92.9 to 98.6) | 77.0 (69.6 to 83.1) | 95.1 (91.6 to 97.2) |
| 0.475 | 69.8 (62.7 to 76.1) | 95.8 (93.4 to 97.4) | 46.7 (30.2 to 63.9) | 96.9 (92.9 to 98.6) | 75.0 (67.5 to 81.3) | 95.5 (92.1 to 97.5) |
| 0.5 | 68.2 (61.0 to 74.5) | 95.8 (93.4 to 97.4) | 46.7 (30.2 to 63.9) | 96.9 (92.9 to 98.6) | 73.0 (65.3 to 79.5) | 95.5 (92.1 to 97.5) |
| 0.525 | 65.9 (58.7 to 72.5) | 95.8 (93.4 to 97.4) | 46.7 (30.2 to 63.9) | 96.9 (92.9 to 98.6) | 70.3 (62.5 to 77.0) | 95.5 (92.1 to 97.5) |
| 0.55 | 62.6 (55.3 to 69.3) | 96.3 (94.0 to 97.8) | 40.0 (24.6 to 57.7) | 97.5 (93.7 to 99.0) | 67.6 (59.7 to 74.6) | 95.9 (92.6 to 97.8) |
| 0.575 | 59.8 (52.5 to 66.7) | 96.6 (94.3 to 98.0) | 36.7 (21.9 to 54.5) | 97.5 (93.7 to 99.0) | 64.9 (56.9 to 72.1) | 96.3 (93.1 to 98.0) |
| 0.6 | 57.0 (49.7 to 64.0) | 97.1 (94.9 to 98.3) | 36.7 (21.9 to 54.5) | 97.5 (93.7 to 99.0) | 61.5 (53.5 to 68.9) | 96.7 (93.7 to 98.3) |
| 0.625 | 50.3 (43.0 to 57.5) | 97.3 (95.2 to 98.5) | 23.3 (11.8 to 40.9) | 97.5 (93.7 to 99.0) | 56.1 (48.0 to 63.8) | 97.1 (94.2 to 98.6) |
| 0.65 | 46.9 (39.8 to 54.2) | 97.6 (95.6 to 98.7) | 23.3 (11.8 to 40.9) | 97.5  (93.7 to 99.0) | 52.0 (44.0 to 59.9) | 97.5 (94.7 to 98.9) |
| 0.675 | 43.6 (36.5 to 50.9) | 98.0 (96.2 to 99.0) | 16.7 (7.34 to 33.6) | 98.1 (94.6 to 99.4) | 49.3 (41.4 to 57.3) | 98.0 (95.3 to 99.1) |
| 0.7 | 39.1 (32.3 to 46.4) | 98.0 (96.2 to 99.0) | 16.7 (7.34 to 33.6) | 98.1 (94.6 to 99.4) | 43.9 (36.2 to 52.0) | 98.0 (95.3 to 99.1) |
| 0.725 | 35.2 (28.6 to 42.4) | 98.0 (96.2 to 99.0) | 16.7 (7.34 to 33.6) | 98.1 (94.6 to 99.4) | 39.2 (31.7 to 47.2) | 98.0 (95.3 to 99.1) |
| 0.75 | 33.0 (26.5 to 40.1) | 98.0 (96.2 to 99.0) | 13.3 (5.31 to 29.7) | 98.1 (94.6 to 99.4) | 37.2 (29.8 to 45.2) | 98.0 (95.3 to 99.1) |
| 0.775 | 30.2 (23.9 to 37.3) | 98.0 (96.2 to 99.0) | 10.0 (3.46 to 25.6) | 98.1 (94.6 to 99.4) | 34.5 (27.3 to 42.4) | 98.0 (95.3 to 99.1) |
| 0.8 | 29.6 (23.4 to 36.7) | 98.8 (97.2 to 99.5) | 10.0 (3.46 to 25.6) | 100.0 (97.6 to 100.0) | 33.8 (26.7 to 41.7) | 98.0 (95.3 to 99.1) |

Youden’s index and associated performance are shown in boldface.
CI, confidence interval; MCI, mild cognitive impairment; MD, mild dementia; NPA, negative percent agreement; PPA, positive percent agreement;
pTau217, tau phosphorylated at threonine 217; SCD, subjective cognitive decline.

**Table S5.** Performance overview at various plasma pTau217 cutoff values with respect to centiloid 40.

| **Cutoff, pg/mL** | **PPA (overall), (95 % CI)** | **NPA (overall), % (95 % CI)** | **PPA (SCD), % (95 % CI)** | **NPA (SCD), %  (95 % CI)** | **PPA (MCI+MD), % (95 % CI)** | **NPA (MCI+MD), % (95 % CI)** |
| --- | --- | --- | --- | --- | --- | --- |
| 0.25 | 95.5 (91.1 to 97.8) | 73.3 (68.9 to 77.3) | 95.7 (79.0 to 99.8) | 78.9 (72.1 to 84.4) | 95.5 (90.6 to 97.9) | 69.8 (63.9 to 75.0) |
| 0.275 | 93.6 (88.7 to 96.5) | 77.7 (73.6 to 81.4) | 91.3 (73.2 to 97.6) | 81.3 (74.7 to 86.5) | 94.0 (88.7 to 96.9) | 75.6 (70.0 to 80.4) |
| 0.3 | 91.1 (85.6 to 94.6) | 81.9 (78.0 to 85.3) | 91.3 (73.2 to 97.6) | 84.9 (78.7 to 89.6) | 91.0 (85.0 to 94.8) | 80.2 (74.9 to 84.6) |
| 0.325 | 88.5 (82.6 to 92.6) | 87.2 (83.8 to 90.1) | 87.0 (67.9 to 95.5) | 91.0 (85.6 to 94.4) | 88.8 (82.4 to 93.1) | 85.3 (80.4 to 89.1) |
| 0.35 | 87.3 (81.1 to 91.6) | 88.6 (85.3 to 91.3) | 82.6 (62.9 to 93.0) | 92.2 (87.1 to 95.4) | 88.1 (81.5 to 92.5) | 86.8 (82.1 to 90.4) |
| **0.358** | **87.3 (81.1 to 91.6)** | **89.6 (86.3 to 92.1)** | **82.6 (62.9 to 93.0)** | **93.4 (88.5 to 96.3)** | **88.1 (81.5 to 92.)** | **87.6 (83.0 to 91.1)** |
| 0.375 | 84.1 (77.5 to 89.0) | 90.5 (87.3 to 92.9) | 69.6 (49.1 to 84.4) | 94.6 (90.0 to 97.1) | 86.6 (79.8 to 91.3) | 88.4 (83.9 to 91.7) |
| 0.4 | 80.9 (74.0 to 86.3) | 91.9 (88.9 to 94.1) | 65.2 (44.9 to 81.2) | 95.8 (91.6 to 97.9) | 83.6 (76.4 to 88.9) | 89.9 (85.6 to 93.0) |
| 0.425 | 78.3 (71.3 to 84.1) | 92.3 (89.4 to 94.5) | 65.2 (44.9 to 81.2) | 95.8 (91.6 to 97.9) | 80.6 (73.1 to 86.4) | 90.7 (86.5 to 93.7) |
| 0.45 | 75.2 (67.9 to 81.3) | 93.0 (90.2 to 95.1) | 60.9 (40.8 to 77.8) | 95.8 (91.6 to 97.9) | 77.6 (69.8 to 83.8) | 91.5 (87.4 to 94.3) |
| 0.475 | 72.6 (65.2 to 79.0) | 93.5 (90.8 to 95.5) | 56.5 (36.8 to 74.4) | 96.4 (92.3 to 98.3) | 75.4 (67.4 to 81.9) | 91.9 (87.9 to 94.6) |
| 0.5 | 70.7 (63.2 to 77.3) | 93.5 (90.8 to 95.5) | 56.5 (36.8 to 74.4) | 96.4 (92.3 to 98.3) | 73.1 (65.1 to 79.9) | 91.9 (87.9 to 94.6) |
| 0.525 | 68.2 (60.5 to 74.9) | 93.5 (90.8 to 95.5) | 56.5 (36.8 to 74.4) | 96.4 (92.3 to 98.3) | 70.1 (61.9 to 77.2) | 91.9 (87.9 to 94.6) |
| 0.55 | 64.3 (56.6 to 71.4) | 94.0 (91.3 to 95.9) | 47.8 (29.2 to 67.0) | 97.0 (93.1 to 98.7) | 67.2 (58.8 to 74.5) | 92.2 (88.3 to 94.9) |
| 0.575 | 61.1 (53.3 to 68.4) | 94.2 (91.6 to 96.0) | 43.5 (25.6 to 63.2) | 97.0 (93.1 to 98.7) | 64.2 (55.8 to 71.8) | 92.6 (88.8 to 95.2) |
| 0.6 | 58.0 (50.1 to 65.4) | 94.7 (92.1 to 96.4) | 43.5 (25.6 to 63.2) | 97.0 (93.1 to 98.7) | 60.4 (52.0 to 68.3) | 93.0 (89.2 to 95.5) |
| 0.625 | 51.0 (43.2 to 58.7) | 95.1 (92.7 to 96.8) | 26.1 (12.5 to 46.5) | 97.0 (93.1 to 98.7) | 55.2 (46.8 to 63.4) | 93.8 (90.2 to 96.1) |
| 0.65 | 47.8 (40.1 to 55.5) | 95.6 (93.2 to 97.2) | 26.1 (12.5 to 46.5) | 97.0 (93.1 to 98.7) | 51.5 (43.1 to 59.8) | 94.6 (91.1 to 96.7) |
| 0.675 | 44.6 (37.0 to 52.4) | 96.3 (94.1 to 97.7) | 21.7 (9.66 to 41.9) | 98.2 (94.8 to 99.4) | 48.5 (40.2 to 56.9) | 95.0 (91.6 to 97.0) |
| 0.7 | 40.8 (33.4 to 48.6) | 96.8 (94.6 to 98.1) | 21.7 (9.66 to 41.9) | 98.2 (94.8 to 99.4) | 44.0 (35.9 to 52.5) | 95.7 (92.5 to 97.6) |
| 0.725 | 36.9 (29.8 to 44.7) | 97.0 (94.9 to 98.2) | 21.7 (9.66 to 41.9) | 98.2 (94.8 to 99.4) | 39.6 (31.7 to 48.0) | 96.1 (93.0 to 97.9) |
| 0.75 | 34.4 (27.4 to 42.1) | 97.0 (94.9 to 98.2) | 17.4 (6.98 to 37.1) | 98.2 (94.8 to 99.4) | 37.3 (29.6 to 45.7) | 96.1 (93.0 to 97.9) |
| 0.775 | 31.8 (25.1 to 39.5) | 97.2 (95.2 to 98.4) | 13.0 (4.54 to 32.1) | 98.2 (94.8 to 99.4) | 35.1 (27.5 to 43.5) | 96.5 (93.5 to 98.2) |
| 0.8 | 31.2 (24.5 to 38.8) | 97.9 (96.1 to 98.9) | 13.0 (4.54 to 32.1) | 100.0 (97.7 to 100.0) | 34.3 (26.8 to 42.7) | 96.5 (93.5 to 98.2) |

Youden’s index and associated performance are shown in boldface.
CI, confidence interval; MCI, mild cognitive impairment; MD, mild dementia; NPA, negative percent agreement; PPA, positive percent agreement;
pTau217, tau phosphorylated at threonine 217; SCD, subjective cognitive decline.

**Table S6.** Performance overview at various plasma pTau217 values with respect to centiloid 50.

| **Cutoff, pg/mL** | **PPA (overall), (95 % CI)** | **NPA (overall), % (95 % CI)** | **PPA (SCD), % (95 % CI)** | **NPA (SCD), %  (95 % CI)** | **PPA (MCI+MD), % (95 % CI)** | **NPA (MCI+MD), % (95 % CI)** |
| --- | --- | --- | --- | --- | --- | --- |
| 0.25 | 96.6 (92.4 to 98.6) | 72.4 (68.1 to 76.4) | 95.2 (77.3 to 99.8) | 78.0 (71.1 to 83.6) | 96.9 (92.2 to 98.8) | 68.9 (63.1 to 74.2) |
| 0.275 | 94.6 (89.8 to 97.3) | 76.8 (72.6 to 80.5) | 90.5 (71.1 to 97.3) | 80.4 (73.7 to 85.7) | 95.3 (90.2 to 97.8) | 74.6 (69.0 to 79.5) |
| 0.3 | 93.3 (88.1 to 96.3) | 81.3 (77.4 to 84.7) | 90.5 (71.1 to 97.3) | 83.9 (77.6 to 88.7) | 93.8 (88.2 to 96.8) | 79.9 (74.7 to 84.3) |
| 0.325 | 90.6 (84.8 to 94.3) | 86.6 (83.1 to 89.4) | 85.7 (65.4 to 95.0) | 89.9 (84.4 to 93.6) | 91.4 (85.3 to 95.1) | 84.8 (80.0 to 88.7) |
| 0.35 | 89.3 (83.3 to 93.3) | 87.9 (84.5 to 90.7) | 81.0 (60.0 to 92.3) | 91.1 (85.8 to 94.5) | 90.6 (84.3 to 94.6) | 86.4 (81.7 to 90.0) |
| **0.358** | **89.3 (83.3 to 93.3)** | **88.8 (85.5 to 91.5)** | **81.0 (60.0 to 92.3)** | **92.3 (87.2 to 95.4)** | **90.6 (84.3 to 94.6)** | **87.1 (82.5 to 90.6)** |
| 0.375 | 85.9 (79.4 to 90.6) | 89.7 (86.6 to 92.3) | 66.7 (45.4 to 82.8) | 93.5 (88.7 to 96.3) | 89.1 (82.5 to 93.4) | 87.9 (83.4 to 91.3) |
| 0.4 | 82.6 (75.7 to 87.8) | 91.1 (88.1 to 93.4) | 61.9 (40.9 to 79.2) | 94.6 (90.1 to 97.2) | 85.9 (78.9 to 90.9) | 89.4 (85.1 to 92.6) |
| 0.425 | 79.9 (72.7 to 85.5) | 91.6 (88.6 to 93.8) | 61.9 (40.9 to 79.2) | 94.6 (90.1 to 97.2) | 82.8 (75.3 to 88.4) | 90.2 (86.0 to 93.2) |
| 0.45 | 77.2 (69.8 to 83.2) | 92.5 (89.6 to 94.6) | 61.9 (40.9 to 79.2) | 95.2 (90.9 to 97.6) | 79.7 (71.9 to 85.7) | 90.9 (86.8 to 93.8) |
| 0.475 | 74.5 (66.9 to 80.8) | 92.9 (90.2 to 95.0) | 57.1 (36.5 to 75.5) | 95.8 (91.7 to 98.0) | 77.3 (69.4 to 83.7) | 91.3 (87.3 to 94.1) |
| 0.5 | 72.5 (64.8 to 79.0) | 92.9 (90.2 to 95.0) | 57.1 (36.5 to 75.5) | 95.8 (91.7 to 98.0) | 75.0 (66.8 to 81.7) | 91.3 (87.3 to 94.1) |
| 0.525 | 69.8 (62.0 to 76.6) | 92.9 (90.2 to 95.0) | 57.1 (36.5 to 75.5) | 95.8(91.7 to 98.0) | 71.9 (63.5 to 78.9) | 91.3 (87.3 to 94.1) |
| 0.55 | 66.4 (58.5 to 73.5) | 93.6  (90.9 to 95.6) | 52.4 (32.4 to 71.7) | 97.0 (93.2 to 98.7) | 68.8 (60.3 to 76.1) | 91.7 (87.7 to 94.4) |
| 0.575 | 63.1 (55.1 to 70.4) | 93.8 (91.2 to 95.7) | 47.6 (28.3 to 67.6) | 97.0 (93.2 to 98.7) | 65.6 (57.0 to 73.3) | 92.0 (88.1 to 94.7) |
| 0.6 | 59.7 (51.7 to 67.3) | 94.3 (91.7 to 96.1) | 47.6 (28.3 to 67.6) | 97.0 (93.2 to 98.7) | 61.7 (53.1 to 69.7) | 92.4 (88.6 to 95.0) |
| 0.625 | 52.3 (44.4 to 60.2) | 94.8 (92.3 to 96.5) | 28.6 (13.8 to 50.0) | 97.0 (93.2 to 98.7) | 56.3 (47.6 to 64.5) | 93.2 (89.5 to 95.6) |
| 0.65 | 49.7 (41.7 to 57.6) | 95.4 (93.1 to 97.0) | 28.6 (13.8 to 50.0) | 97.0 (93.2 to 98.7) | 53.1 (44.5 to 61.6) | 94.3 (90.8 to 96.5) |
| 0.675 | 46.3 (38.5 to 54.3) | 96.1 (93.9 to 97.6) | 23.8 (10.6 to 45.1) | 98.2 (94.9 to 99.4) | 50.0 (41.5 to 58.5) | 94.7 (91.3 to 96.8) |
| 0.7 | 42.3 (34.6 to 50.3) | 96.6 (94.4 to 97.9) | 23.8 (10.6 to 45.1) | 98.2 (94.9 to 99.4) | 45.3 (37.0 to 53.9) | 95.5 (92.2 to 97.4) |
| 0.725 | 38.3 (30.8 to 46.3) | 96.8 (94.7 to 98.1) | 23.8 (10.6 to 45.1) | 98.2 (94.9 to 99.4) | 40.6 (32.5 to 49.3) | 95.8 (92.7 to 97.7) |
| 0.75 | 35.6 (28.3 to 43.5) | 96.8 (94.7 to 98.1) | 19.0 (7.67 to 40.0) | 98.2 (94.9 to 99.4) | 38.3 (30.3 to 46.9) | 95.8 (92.7 to 97.7) |
| 0.775 | 32.9 (25.9 to 40.8) | 97.0 (95.0 to 98.3) | 14.3 (4.98 to 34.6) | 98.2 (94.9 to 99.4) | 35.9 (28.1 to 44.5) | 96.2 (93.2 to 97.9) |
| 0.8 | 32.2 (25.2 to 40.1) | 97.7 (95.9 to 98.8) | 14.3 (4.98 to 34.6) | 100.0 (97.8 to 100.0) | 35.2 (27.4 to 43.8) | 96.2 (93.2 to 97.9) |

Youden’s index and associated performance are shown in boldface.
CI, confidence interval; MCI, mild cognitive impairment; MD, mild dementia; NPA, negative percent agreement; PPA, positive percent agreement;
pTau217, tau phosphorylated at threonine 217; SCD, subjective cognitive decline.

**Table S7.** Contingency table of plasma pTau217 versus PET centiloid-based classification at different cutoffs.

|  |  | **Positive, *n* (%)** | **Negative, *n* (%)** | **Sum, *n* (%)** | **LR (95 % CI)** | **PV, % (95 % CI)** |
| --- | --- | --- | --- | --- | --- | --- |
| Centiloid 24.1 status at 33.2 % prevalence;  lower cutoff=0.234 pg/mL, upper cutoff=0.317 pg/mL | Positive | 160 (82.1) | 39 (9.92) | 199 (33.8) | 8.27 (6.09 to 11.2) | 80.4 (75.1 to 84.8) |
|  | Indeterminate | 16 (8.21) | 69 (17.6) | 85 (14.5) | 0.467 (0.279 to 0.783) | 18.8 (12.2 to 28.0) |
|  | Negative | 19 (9.74) | 285 (72.5) | 304 (51.7) | 0.134 (0.0873 to 0.207) | 6.25 (4.15 to 9.31) |
|  | Total | 195 | 393 | 588 (100) | — | — |
| Centiloid 30 status at 30.4 % prevalence;  lower cutoff=0.291 pg/mL, upper cutoff=0.318 pg/mL | Positive | 157 (87.7) | 40 (9.78) | 197 (33.5) | 8.97 (6.65 to 12.1) | 79.7 (74.4 to 84.1) |
|  | Indeterminate | 5 (2.79) | 29 (7.09) | 34 (5.78) | 0.394 (0.155 to 1.00) | 14.7 (6.35 to 30.5) |
|  | Negative | 17 (9.50) | 340 (83.1) | 357 (60.7) | 0.114 (0.0725 to 0.180) | 4.76 (3.08 to 7.30) |
|  | Total | 179 | 409 | 588 (100) | — | — |
| Centiloid 40 status at 26.7 % prevalence;  lower cutoff=0.305 pg/mL, upper cutoff=0.364 pg/mL | Positive | 133 (84.7) | 43 (9.98) | 176 (29.9) | 8.49 (6.35 to 11.4) | 75.6 (69.8 to 80.5) |
|  | Indeterminate | 10 (6.37) | 33 (7.66) | 43 (7.31) | 0.832 (0.420 to 1.65) | 23.3 (13.3 to 37.5) |
|  | Negative | 14 (8.92) | 355 (82.4) | 369 (62.8) | 0.108 (0.0655 to 0.179) | 3.79 (2.33 to 6.12) |
|  | Total | 157 | 431 | 588 (100) | — | — |

| Centiloid 50 status at 25.3 % prevalence;  lower cutoff=0.337 pg/mL, upper cutoff=0.387 pg/mL | Positive | 128 (85.9) | 43 (9.79) | 171 (29.1) | 8.77 (6.55 to 11.7) | 74.9 (69.0 to 79.9) |
| --- | --- | --- | --- | --- | --- | --- |
|  | Indeterminate | 7 (4.70) | 12 (2.73) | 19 (3.23) | 1.72 (0.689 to 4.28) | 36.8 (19.0 to 59.3) |
|  | Negative | 14 (9.40) | 384 (87.5) | 398 (67.7) | 0.107 (0.0652 to 0.177) | 3.52 (2.16 to 5.67) |
|  | Total | 149 | 439 | 588 (100) | — | — |

CI, confidence interval; LR, likelihood ratio; PET, positron emission tomography; pTau217, tau phosphorylated at threonine 217; PV, predictive value.

**Table S8.** Contingency table of plasma pTau217 versus PET centiloid-based classification at different cutoffs for individuals diagnosed with SCD only.

|  |  | **Positive, *n* (%)** | **Negative, *n* (%)** | **Sum, *n* (%)** | **LR (95 % CI)** | **PV, % (95 % CI)** |
| --- | --- | --- | --- | --- | --- | --- |
| Centiloid 24.1 status at 18.0 % prevalence;  lower cutoff=0.234 pg/mL, upper cutoff=0.317 pg/mL | Positive | 26 (76.5) | 11 (7.10) | 37 (19.6) | 10.8 (5.92 to 19.6) | 70.3 (56.5 to 81.1) |
|  | Indeterminate | 3 (8.82) | 28 (18.1) | 31 (16.4) | 0.488 (0.158 to 1.51) | 9.68 (3.34 to 24.9) |
|  | Negative | 5 (14.7) | 116 (74.8) | 121 (64.0) | 0.197 (0.0870 to 0.444) | 4.13 (1.87 to 8.87) |
|  | Total | 34 | 155 | 189 (100) | — | — |
| Centiloid 30 status at 15.9 % prevalence;  lower cutoff=0.291 pg/mL, upper cutoff=0.318 pg/mL | Positive | 25 (83.3) | 11 (6.92) | 36 (19.0) | 12.0 (6.66 to 21.8) | 69.4 (55.7 to 80.4) |
|  | Indeterminate | 2 (6.67) | 10 (6.29) | 12 (6.35) | 1.06 (0.244 to 4.60) | 16.7 (4.41 to 46.4) |
|  | Negative | 3 (10.0) | 138 (86.8) | 141 (74.6) | 0.115 (0.0393 to 0.338) | 2.13 (0.736 to 5.99) |
|  | Total | 30 | 159 | 189 (100) | — | — |
| Centiloid 40 status at 12.2 % prevalence;  lower cutoff=0.305 pg/mL, upper cutoff=0.364 pg/mL | Positive | 17 (73.9) | 10 (6.02) | 27 (14.3) | 12.3 (6.42 to 23.5) | 63.0 (47.1 to 76.5) |
|  | Indeterminate | 4 (17.4) | 14 (8.43) | 18 (9.52) | 2.06 (0.742 to 5.73) | 22.2 (9.32 to 44.3) |
|  | Negative | 2 (8.70) | 142 (85.5) | 144 (76.2) | 0.102 (0.0270 to 0.383) | 1.39 (0.373 to 5.04) |
|  | Total | 23 | 166 | 189 (100) | — | — |

| Centiloid 50 status at 11.1 % prevalence;  lower cutoff=0.337 pg/mL, upper cutoff=0.387 pg/mL | Positive | 14 (66.7) | 9 (5.36) | 23 (12.2) | 12.4 (6.16 to 25.2) | 60.9 (43.5 to 75.9) |
| --- | --- | --- | --- | --- | --- | --- |
|  | Indeterminate | 4 (19.0) | 7 (4.17) | 11 (5.82) | 4.57 (1.46 to 14.3) | 36.4 (15.4 to 64.2) |
|  | Negative | 3 (14.3) | 152 (90.5) | 155 (82.0) | 0.158 (0.0553 to 0.451) | 1.94 (0.687 to 5.33) |
|  | Total | 21 | 168 | 189 (100) | — | — |

CI, confidence interval; LR, likelihood ratio; PET, positron emission tomography; pTau217, tau phosphorylated at threonine 217; PV, predictive value;
SCD, subjective cognitive decline.

**Table 9.** Contingency table of plasma pTau217 versus PET centiloid-based classification at different cutoffs for individuals diagnosed with MCI and MD only.

|  |  | **Positive, *n* (%)** | **Negative, *n* (%)** | **Sum, *n* (%)** | **LR (95 % CI)** | **PV, % (95 % CI)** |
| --- | --- | --- | --- | --- | --- | --- |
| Centiloid 24.1 status at 40.8 % prevalence;  lower cutoff=0.234 pg/mL, upper cutoff=0.317 pg/mL | Positive | 133 (83.1) | 27 (11.6) | 160 (40.8) | 7.14 (4.98 to 10.3) | 83.1 (77.4 to 87.6) |
|  | Indeterminate | 13 (8.13) | 41 (17.7) | 54 (13.8) | 0.460 (0.255 to 0.830) | 24.1 (14.9 to 36.4) |
|  | Negative | 14 (8.75) | 164 (70.7) | 178 (45.4) | 0.124 (0.0745 to 0.206) | 7.87 (4.89 to 12.4) |
|  | Total | 160 | 232 | 392 (100) | — | — |
| Centiloid 30 status at 37.8 % prevalence;  lower cutoff=0.291 pg/mL, upper cutoff=0.318 pg/mL | Positive | 131 (88.5) | 28 (11.5) | 159 (40.6) | 7.71 (5.42 to 11.0) | 82.4 (76.7 to 86.9) |
|  | Indeterminate | 3 (2.03) | 19 (7.79) | 22 (5.61) | 0.260 (0.0784 to 0.865) | 13.6 (4.54 to 34.4) |
|  | Negative | 14 (9.46) | 197 (80.7) | 211 (53.8) | 0.117 (0.0709 to 0.194) | 6.64 (4.12 to 10.5) |
|  | Total | 148 | 244 | 392 (100) | — | — |
| Centiloid 40 status at 34.2 % prevalence;  lower cutoff=0.305 pg/mL, upper cutoff=0.364 pg/mL | Positive | 116 (86.6) | 31 (12.0) | 147 (37.5) | 7.20 (5.14 to 10.1) | 78.9 (72.8 to 84.0) |
|  | Indeterminate | 6 (4.48) | 19 (7.36) | 25 (6.38) | 0.608 (0.249 to 1.49) | 24.0 (11.4 to 43.6) |
|  | Negative | 12 (8.96) | 208 (80.6) | 220 (56.1) | 0.111 (0.0645 to 0.191) | 5.45 (3.24 to 9.03) |
|  | Total | 134 | 258 | 392 (100) | — | — |

| Centiloid 50 status at 32.7 % prevalence;  lower cutoff=0.337 pg/mL, upper cutoff=0.387 pg/mL | Positive | 114 (89.1) | 32 (12.1) | 146 (37.2) | 7.35 (5.28 to 10.2) | 78.1 (71.9 to 83.2) |
| --- | --- | --- | --- | --- | --- | --- |
|  | Indeterminate | 3 (2.34) | 5 (1.89) | 8 (2.04) | 1.24 (0.300 to 5.10) | 37.5 (12.7 to 71.2) |
|  | Negative | 11 (8.59) | 227 (86.0) | 238 (60.7) | 0.0999 (0.0567 to 0.176) | 4.62 (2.67 to 7.87) |
|  | Total | 128 | 264 | 392 (100) | — | — |

CI, confidence interval; LR, likelihood ratio; MCI, mild cognitive impairment; MD, mild dementia; PET, positron emission tomography;
pTau217, tau phosphorylated at threonine 217; PV, predictive value.

**Fig. S1.** Centiloid distribution by clinical diagnosis.


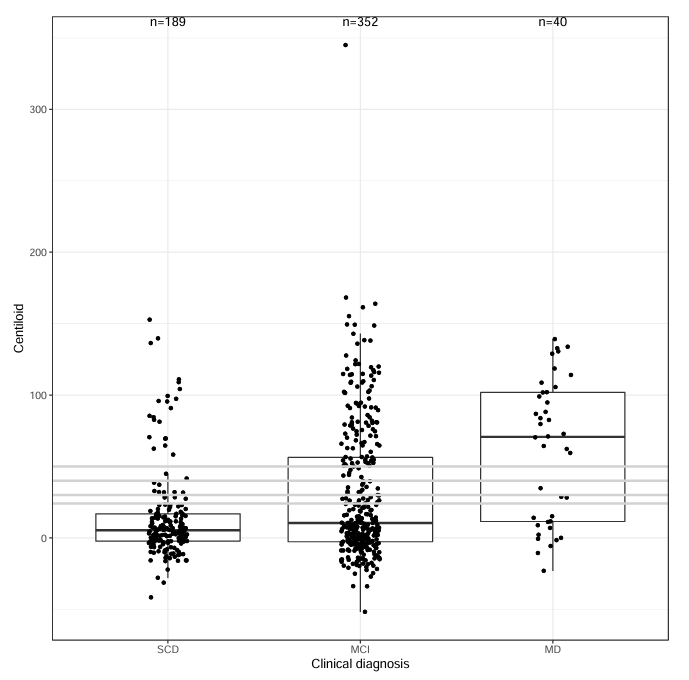


The applied centiloid cutoffs are shown by the grey lines; 24.1, 30, 40, and 50.
MCI, mild cognitive impairment; MD, mild dementia; SCD, subjective cognitive decline.

**Fig. S2**. ROC-AUC analyses for plasma pTau217 with respect to varying centiloid-based classification cutoffs.


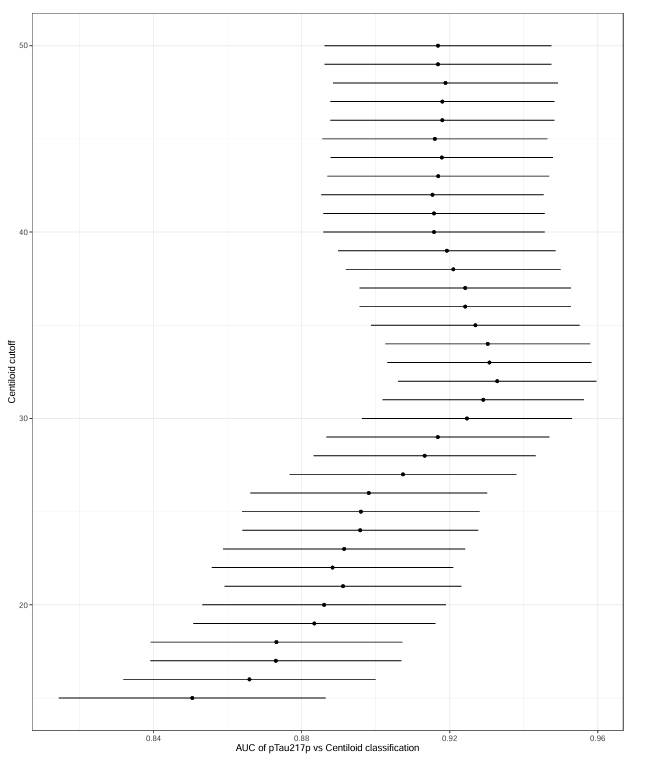


AUC, area under the curve; pTau217, tau phosphorylated at threonine 217; ROC, receiver operating characteristic.

**Fig. S3.** Cumulative distribution analysis illustrating the PPA and NPA of the prototype pTau217 plasma immunoassay with respect to centiloid-based classification at selected cutoffs.


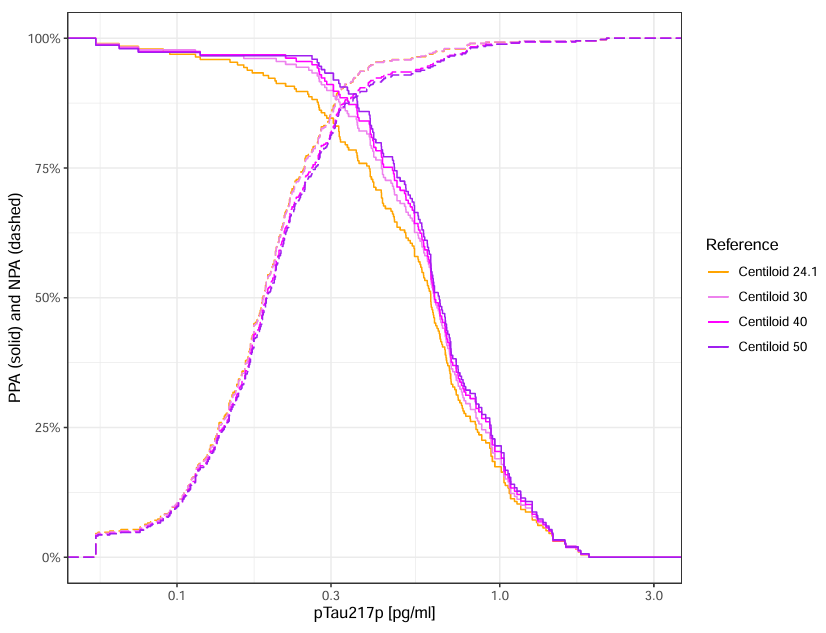


NPA, negative percent agreement; PPA, positive percent agreement; pTau217, tau phosphorylated at threonine 217.

**Fig. S4**. Distribution of plasma pTau217 concentrations by age subgroup with respect to centiloid cutoff 30 using a double cutoff approach set at 90 % PPA and 90 % NPA. The box itself represents the interquartile range. The median is represented by a solid horizontal line within the box. The whiskers extend from the box to the most extreme values, excluding outliers. The double cutoffs are represented by grey lines.


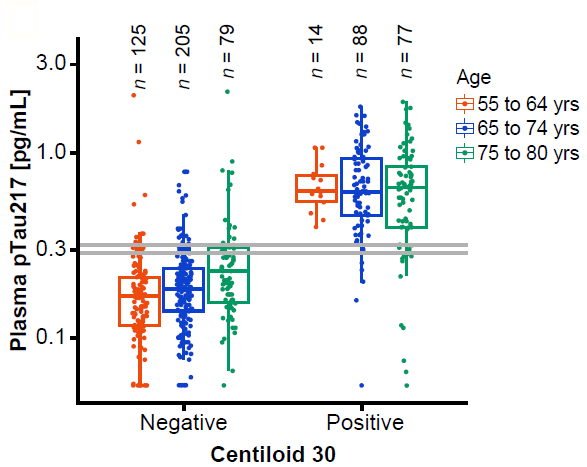


NPA, negative percent agreement; PPA, positive percent agreement; pTau217, tau phosphorylated
at threonine 217; yrs, years.

**Fig. S5**. Distribution of plasma pTau217 concentrations by participant sex with respect to centiloid cutoff 30 using a double cutoff approach set at 90 % PPA and 90 % NPA. The box itself represents the interquartile range. The median is represented by a solid horizontal line within the box. The whiskers extend from the box to the most extreme values, excluding outliers. The double cutoffs are represented by grey lines.


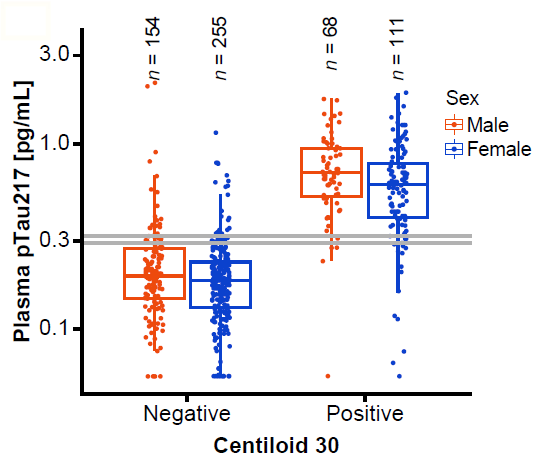


NPA, negative percent agreement; PPA, positive percent agreement; pTau217, tau phosphorylated
at threonine 217.
